# The Myelin‐Weighted Connectome in Parkinson's Disease

**DOI:** 10.1002/mds.28891

**Published:** 2021-12-22

**Authors:** Tommy Boshkovski, Julien Cohen‐Adad, Bratislav Misic, Isabelle Arnulf, Jean‐Christophe Corvol, Marie Vidailhet, Stéphane Lehéricy, Nikola Stikov, Matteo Mancini

**Affiliations:** ^1^ NeuroPoly Lab, Polytechnique Montréal Montréal Quebec Canada; ^2^ Mila – Quebec AI Institute Montréal Quebec Canada; ^3^ Functional Neuroimaging Unit, Centre de Recherche de l'Institut Universitaire de Gériatrie de Montréal Montréal Quebec Canada; ^4^ Montreal Neurological Institute Montréal Quebec Canada; ^5^ Sorbonne Université, Paris Brain Institute – ICM, INSERM, CNRS, Assistance Publique Hôpitaux de Paris, Hôpital Pitié‐Salpêtrière Paris France; ^6^ Montreal Heart Institute Montréal Quebec Canada; ^7^ Department of Neuroscience Brighton and Sussex Medical School, University of Sussex Brighton United Kingdom; ^8^ Cardiff University Brain Research Imaging Centre (CUBRIC), Cardiff University Cardiff United Kingdom

**Keywords:** myelin, connectome, Parkinson's disease

## Abstract

**Background:**

Even though Parkinson's disease (PD) is typically viewed as largely affecting gray matter, there is growing evidence that there are also structural changes in the white matter. Traditional connectomics methods that study PD may not be specific to underlying microstructural changes, such as myelin loss.

**Objective:**

The primary objective of this study is to investigate the PD‐induced changes in myelin content in the connections emerging from the basal ganglia and the brainstem. For the weighting of the connectome, we used the longitudinal relaxation rate as a biologically grounded myelin‐sensitive metric.

**Methods:**

We computed the myelin‐weighted connectome in 35 healthy control subjects and 81 patients with PD. We used partial least squares to highlight the differences between patients with PD and healthy control subjects. Then, a ring analysis was performed on selected brainstem and subcortical regions to evaluate each node's potential role as an epicenter for disease propagation. Then, we used behavioral partial least squares to relate the myelin alterations with clinical scores.

**Results:**

Most connections (~80%) emerging from the basal ganglia showed a reduced myelin content. The connections emerging from potential epicentral nodes (substantia nigra, nucleus basalis of Meynert, amygdala, hippocampus, and midbrain) showed significant decrease in the longitudinal relaxation rate (*P* < 0.05). This effect was not seen for the medulla and the pons.

**Conclusions:**

The myelin‐weighted connectome was able to identify alteration of the myelin content in PD in basal ganglia connections. This could provide a different view on the importance of myelination in neurodegeneration and disease progression. © 2021 The Authors. *Movement Disorders* published by Wiley Periodicals LLC on behalf of International Parkinson and Movement Disorder Society

## Introduction

Parkinson's disease (PD) is a neurodegenerative disorder that is characterized by motor and nonmotor symptoms. PD is considered to be a disease that mainly affects the gray matter, specifically the dopaminergic neurons that affect motor function. In addition, this disease has been characterized by the accumulation of misfolded ɑ‐synuclein proteins across the brain.^1^, ^2^, ^3^, ^4^ It has also been noted that the myelination status and the size of the axons could play an important role in neurodegeneration in PD.^5^

A well‐established model of PD suggests that the disease progression (ie, neurodegeneration) follows a topological sequence.^6^ According to the Braak hypothesis, there are six stages of disease progression. The disease first affects the lower part of the brainstem and then spreads to the medulla oblongata, the pons, the midbrain, the mesocortex, and finally, the neocortex. An alternative hypothesis for the disease spreading has also been proposed, according to which the disease first starts in the cortex and then spreads to the rest of the brain.^7^, ^8^ It has been recently suggested that neurodegeneration in patients with PD with rapid eye movement sleep behavior disorder (RBD; PDRBD) follows the Braak model (“bottom‐up”) of disease progression,^9^, ^10^ whereas neurodegeneration in patients with PD without RBD (PDnonRBD) follows the alternative “top‐down” model (from cortex to brainstem).^10^, ^11^

Like other neurodegenerative disorders, one possible hypothesis of PD progression is that it progresses by means of a prion‐like mode of transmission throughout the brain.^12^, ^13^, ^14^, ^15^, ^16^ This mechanism of transmission is characterized by the transneuronal spread of misfolded ɑ‐synuclein. In this manner, the pathology can travel from one brain region to another via interconnected neural pathways. This is analogous to how infectious diseases spread through person‐to‐person contact. Hence several studies^12^, ^17^ have used spreading models, such as the susceptible‐infected‐removed model or the network‐diffusion model, to investigate how the degeneration propagates through the brain.

Magnetic resonance imaging (MRI) has made it possible to investigate the neurodegenerative patterns of PD *in vivo*.^18^ In particular, a number of diffusion‐weighted imaging (DWI) studies have demonstrated alterations in brain network architecture in various cortical and subcortical systems. Moreover, changes in tensor metrics, such as radial diffusivity and fractional anisotropy, in nigrostriatal fibers are related to the extent of motor deficits present in patients with PD.^19^ White matter abnormalities are routinely found in the white matter of the frontal and parietal lobes.^20^, ^21^ White matter microstructural damage in patients with PD who do not exhibit cognitive impairment has also been found to occur before gray matter atrophy can be detected.^22^ Although DWI has repeatedly identified white matter abnormalities in PD, DWI metrics are not specific to a single microstructural property and can be affected by different pathological mechanisms (eg, neuronal/myelin loss). Only recently have researchers started to investigate whether PD affects myelin in white matter. In the study of Dean et al.,^23^ the authors performed voxel‐wise comparisons of myelin‐sensitive MRI metrics (myelin water fraction [MWF], longitudinal relaxation rate [R1], transverse relaxation rate [R2]) and found myelin alterations in the frontal and temporal white matter (R2, MWF), as well as in the thalamus (R1). Another study^24^ used the MWF to investigate the alterations in myelin content in 20 different white matter regions of interest (ROIs). They did not find significant differences between the patients with PD and healthy control subjects (HCs), but they did find that the MWF was negatively correlated with clinical scores.

Compared with previous studies^23^, ^24^ that focused on investigating voxel‐wise demyelination or considering specific white matter tracts, in this article we took a whole‐brain connectomics approach to investigate the effect of PD on myelin in the white matter. This approach leverages network models and graph theory to characterize the brain structure in terms of connectivity patterns.^25^ As a result, we are able not only to compare patients and healthy subjects in a data‐driven fashion but also to test the role of potential epicenters for the disease. There is a growing interest in evaluating pathology for which myelin‐specific changes in brain connectivity are suspected.^26^, ^27^, ^28^ Several myelin‐sensitive metrics have been proposed, including R1.^29^, ^30^ For this study, we chose R1 because it has been shown to be highly correlated with myelin^31^, ^32^ in a broad range of pathologies. We then performed multivariate statistical analysis to identify (1) connections that differentiate between patients with PD and HC in terms of myelination status, and (2) connections that correlate with Movement Disorder Society Unified Parkinson's Disease Rating Scale (MDS‐UPDRS) Part III clinical score. Furthermore, we tested the hypothesis that most of the alteration of the myelin is concentrated in the connections emerging from the subcortical regions.

## Subjects and Methods

### Subjects

Thirty‐five HCs (12 females/23 males, mean age ± SD: 61.2 ± 9.16 years) and 81 patients with PD (52 females/29 males, mean age ± SD: 61.6 ± 9.6 years) participated in this study. The study was approved by the local ethics committee (CPP Paris VI, RCB: 2009‐A00922‐55). All subjects provided written informed consent.

### Clinical Assessment

The subjects were evaluated using the Hoehn and Yahr staging.^33^ For all subjects, the motor disability was assessed in the *off* state, following 12 hours of withdrawal of dopaminergic treatment, using the MDS‐UPDRD Part III. Also, the subjects were assessed for their cognitive abilities using the Mini‐Mental State Examination (MMSE),^34^ Montreal Cognitive Assessment,^35^ and Mattis Dementia Rating Scale.^36^

The PD patients were also evaluated for presence of RBD using interviews and video polysomnography, following international criteria by the American Academy of Sleep Medicine.^37^ Therefore, the PD patients were subdivided into two groups: PDRBD, which consisted of 22 patients; and PDnonRBD, which included 59 patients.

The demographic data and the clinical scores were compared between the groups (Table 1) using a Kruskal–Wallis nonparametric test. The gender was compared with a χ^2^ test. Significant differences were observed for the MDS‐UPDRS *off* (*P* = 6.95E−17) score, Hoehn and Yahr score (*P* = 4.71E−25), and MMSE score (*P* = 0.025) between HCs and PD patients. Also, there was a significant difference in the disease duration between the PDnonRBD and PDRBD groups; ie, the PDRBD group had 6.8 months longer disease duration than the PDnonRBD group (*P* = 0.017).

**TABLE 1:** Demographic data

	PD			HC	HC vs. PD, *P* value	RBD vs. non‐RBD, *P* value
	Total	PDnonRBD	PDRBD			
n	81	59	22	35	–	–
Gender, F/M	52/29	37/22	15/7	12/23	**2.945E−3**	0.65
Age, mean ± SD (y)	61.6 ± 9.6	60.83 ± 10.21	63.86 ± 7.47	61.2 ± 9.16	0.16	0.34
Disease duration, mean ± SD (mo)	18.95 ± 12.54	**17.11 ± 12.69**	**23.91 ± 10.89**	**–**	**–**	**0.017**
Levodopa equivalent daily dose, mean ± SD	–	318 ± 318	393.4 ± 151.8	–	–	–
MDS‐UPDRS *off*, mean ± SD	**30.53 ± 8.02**	31.07 ± 8.24	29.09 ± 7.4	**5.14 ± 5.15**	**6.95E−17**	0.33
MDS‐UPDRS *on*, mean ± SD	26.61 ± 7.82	25.62 ± 10.47	24.4 ± 6.76	–	–	0.34
H&Y score, mean ± SD	**2.02 ± 0.22**	2 ± 0.19	2.09 ± 0.29	**0 ± 0**	**4.71E−25**	0.1
MMSE score, mean ± SD	**28.96 ± 1.28**	28.9 ± 1.36	29.14 ± 1.08	**29.48 ± 0.78**	**0.025**	0.47
MoCA score, mean ± SD	27.62 ± 2.26	27.61 ± 2.2	27.64 ± 2.48	28.03 ± 1.48	0.66	0.70
MATTIS score, mean ± SD	138.86 ± 4.92	138.71 ± 5.25	139.27 ± 3.96	139.88 ± 3.85	0.51	0.97

The demographic data between the groups were compared with a Kruskal–Wallis test, and the *P* values are reported. For the gender comparison, a χ^2^ test was used. The significant results are highlighted in bold.

PD, Parkinson's disease; PDnonRBD, PD without rapid eye movement sleep behavior disorder; PDRBD, PD with rapid eye movement sleep behavior disorder; HC, healthy control subject; RBD, rapid eye movement sleep behavior disorder; MDS‐UPDRS, Movement Disorder Society Unified Parkinson's Disease Rating Scale; H&Y, Hoehn and Yahr; MMSE, Mini‐Mental State Examination; MoCA, Montreal Cognitive Assessment; MATTIS, Mattis Dementia Rating Scale.

### MRI Data Acquisition

Each of the participants was scanned on a 3T SIEMENS Prisma Scanner following a multimodal acquisition protocol. The protocol for each subject included: (1) three‐shell DWI sequence (repetition time = 10,400 ms; echo time = 59 ms; voxel size = 1.7 × 1.7 × 1.7 mm^3^; gradient directions [per shell] = 64, 32, and 8 at, respectively, b = 2500, 700, 300 s/mm^2^; nondiffusion [b0] images per shell = 6, 6, and 2); (2) magnetization‐prepared 2 rapid acquisition gradient echoes (MP2RAGE) sequence to estimate the R1 (repetition time = 5000 ms; echo time = 2.98 ms; flip angles = 4–5 degrees; inversion time = 700/2700 ms; field of view = 256 × 232 mm; voxel size = 1 × 1 × 1 mm^3^).

### R1 Map Reconstruction

The R1 map was calculated using the qMRLab software tool.^38^ A UNI image is used to reconstruct the map. The UNI image is obtained with a combination of two gradient echo images with different inversion times (INV1 and INV2),^39^ produced with the MP2RAGE protocol,^39^ with different flip angles and with different inversion times. The benefit of using the unified T1‐weighted image is that it is free from proton density and T2* contrast.

### Data Preprocessing

The T1‐weighted images for each subject were first denoised using a robust noise background removal tool^40^ as implemented in https://github.com/JosePMarques/MP2RAGE‐related‐scripts/commit/94de0cb236ba49ffcd47120dd73805779a0330da, and N4 bias field correction (version 2.2)^41^ was applied. After the denoising and the bias correction, the images were processed using FreeSurfer 6.0^42^ to segment the different brain tissues and to parcellate the brain using the Desikan–Killiany atlas.^43^

We used the following additional ROIs to cover all the structures of interest for PD: (1) the midbrain and the pons, as segmented from FreeSurfer^44^; (2) the posterior part of medulla oblongata, which was manually segmented^45^; (3) the locus coeruleus (LC), which was segmented semiautomatically as previously described^46^, ^47^; (4) the nucleus basalis of Meynert (NBM), which was segmented manually using T1‐ and T2‐weighted images; and (5) the substantia nigra (SN), obtained from the atlas of the basal ganglia^48^ and nonlinearly registered to each subject using ANTs 3.0 (http://stnava.github.io/ANTs/). We also made sure that the SN ROI did not overlap with the midbrain ROI.

The diffusion data were preprocessed using MRTrix 3.0.^49^ The pipeline for the diffusion preprocessing is described in Boshkovski et al.^29^ In brief, the diffusion images were first denoised^50^, ^51^ and then corrected for Gibbs ringing artifacts^52^ and B1 field inhomogeneity. Then the images were also corrected for motion^53^ and inhomogeneity distortions^54^ using the FSL's eddy and topup tools, relying on b0 images acquired with reverse‐phase encoding. The tractogram for each subject was reconstructed deterministically using multi‐tissue constrained spherical deconvolution^55^ and anatomically constrained tractography.^56^

### Myelin‐Weighted Networks

The structural connectome for each subject was reconstructed using the Desikan–Killiany parcellation and the reconstructed tractograms. A weight was assigned to each connection in the connectome. The weight was calculated as the median R1 value along the bundle of streamlines between each pair of regions. Then, for each group, a group‐connectome was constructed by taking the median across subjects of the connections that were present in at least 50% of the subjects.^57^ As a complementary approach to the R1‐weighted connectome, we also used diffusion‐based measures as a term of comparison for our results. More details are provided in Appendix S1.

### Partial Least Squares Analysis

First, we wanted to characterize the differences between PD patients and HCs in the whole brain in a data‐driven fashion. Therefore, we used partial least squares (PLS) analysis, which is a multivariate statistical technique used to relate two sets of variables to each other.^58^, ^59^, ^60^, ^61^ The first set of variables represents the weights of the connections, while the second set represents the experiment design. More details about the analysis are provided in Appendix S1.

In this study, we used mean‐centering PLS to compare the HC group with PD groups (HC vs. PD, HC vs. PDnonRBD, HC vs. PDRBD) and behavioral PLS to relate connectivity patterns with clinical scores (MDS‐UPDRS Part III, MMSE, Montreal Cognitive Assessment, Mattis Dementia Rating Scale) for the PD group. The PLS analysis tries to find linear combinations of variables in both sets that maximally covary with each other. For the mean centering, we aimed to find an optimal contrast between HC and PD groups, as well as connectivity patterns that maximally covary with this contrast. In contrast, for the behavioral PLS, we aimed to find a relationship between the connectivity pattern and behavioral (clinical) scores. In this analysis, the mean‐centered and behavioral data matrices are subjected to singular value decomposition, which outputs the mutually orthogonal latent variables (LVs).^62^

The statistical significance of the LVs was estimated using permutation tests (500 permutations). The permutation tests randomly reordered the subjects in the original data matrices while ignoring their group assignments. Then, from the permuted data matrices, we calculated covariance matrices, which were then subjected to singular value decomposition. Because the singular values reflect the degree of the statistical relationship of the LVs, the *P* values were computed as the proportion of the number of times the singular values were greater than the singular values of the original covariance matrix.

To check the reliability of each individual connection, we conducted a bootstrap resampling with 500 bootstraps. From the bootstrap distribution, a standard error was estimated for each connection, that corresponds to the stability of the connection. Afterward, a bootstrap ratio (BSR) was calculated for each connection by dividing the connection's weight (from the singular vectors) by its bootstrap‐estimated standard error. The BSRs were then thresholded at values that corresponded to 99% confidence intervals to extract the most reliable connections. To assess the influence of gender and age on the mean‐centering PLS analysis, we computed the related Pearson correlation coefficients with the LVs.

### Connectivity Ring Analysis

**FIG 1:**
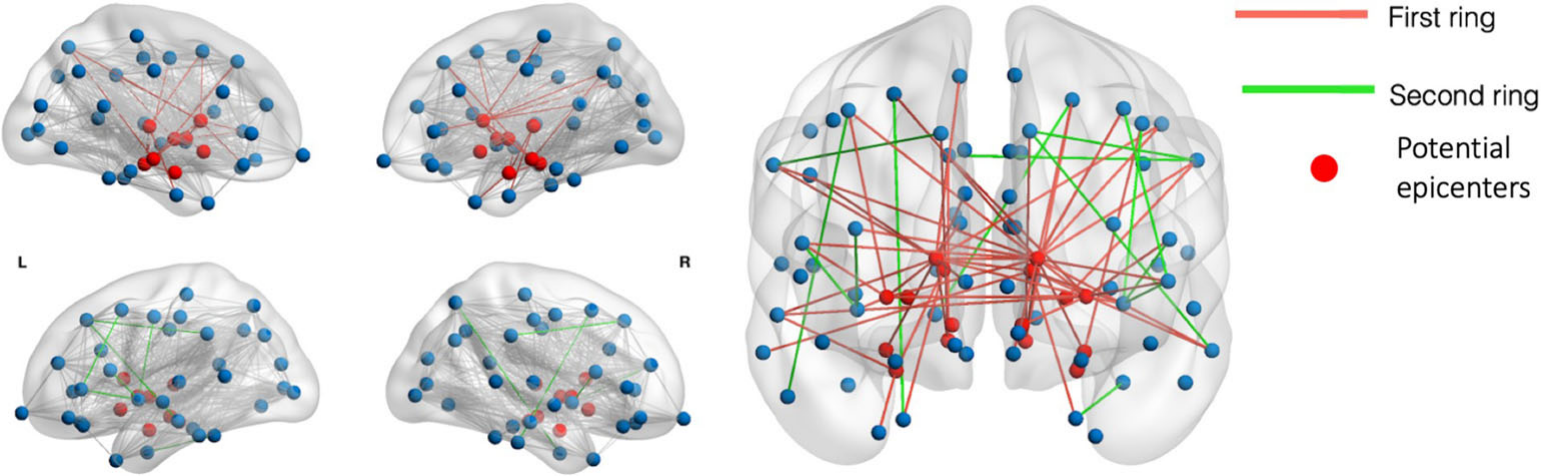
Example representation of the first and second rings. The nodes in red represent the potential epicenters. The connections in red are first ring connections, ie, connections emerging from the epicenters, while the connections in green are second ring connections, ie, connections of the first ring nodes with the second ring nodes. [Color figure can be viewed at wileyonlinelibrary.com]

As a second step, we wanted to investigate the role of specific ROIs in the myelin alterations. Therefore, a connectivity ring analysis^63^ was performed to assess the hypothesis that the connections emerging from the disease epicenters would show greater structural damage in the early stages of the disease. Eight groups of nodes (four subcortical: bilateral SN,^64^ NBM,^65^ hippocampus,^66^ and amygdala^67^; and four brainstem: medulla,^45^ pons,^68^ midbrain,^69^ and LC^70^) were considered as potential epicenter nodes from where the disease would spread. We chose these regions from the relevant literature.^10^ For each potential epicenter, two sets of nodes, denoted as rings, were defined using the information provided by tractography (Fig. 1). The first ring consisted of nodes that were directly connected to one of the epicenter nodes (ie, a set of streamlines intersecting both nodes existed; see Appendix S1), while the second ring was composed of nodes indirectly connected to the epicenter nodes through a single node from the first ring. Then, for each subject, we calculated the median R1 value of the connections for each of the rings. The median R1 value of the rings between the groups (PD and HC) was compared using analysis of covariance while controlling for age and sex, and corrected for multiple comparisons using false discovery rate. To characterize potential differences in myelin content within the epicenters themselves, for each epicenter we computed the median R1 value. We then compared the related distributions for HC and PD groups using two‐sample *t* tests.

## Results

**FIG 2:**
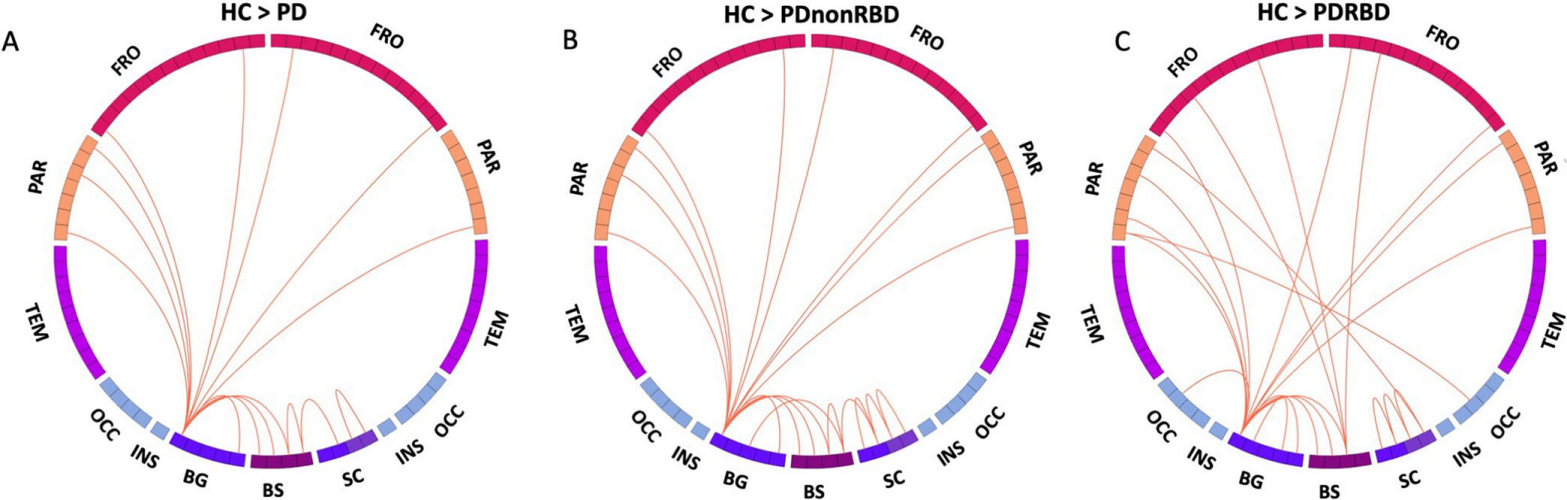
Connectogram of the multivariate connectivity pattern obtained with mean‐centering partial least squares, composed of the connections that maximally covary between the groups. These connections showed decreased longitudinal relaxation rate (R1) in the Parkinson's disease (PD) groups compared with the healthy control (HC) group. Most of the connections associated with a significant HC/PD difference are emerging from the basal ganglia (BG). BS, brainstem; FRO, frontal lobe; OCC, occipital lobe; PAR, parietal lobe; SC, subcortical regions, including amygdala, hippocampus, thalamus, and nucleus basalis of Meynert; TEM, temporal lobe. [Color figure can be viewed at wileyonlinelibrary.com]

To assess the connectome differences between the PD and HC participants, we performed a PLS analysis. This type of analysis allowed us to identify the connections that maximally and reliably (BSR > 2.56) covaried between the two groups. The mean‐centered PLS analysis showed that most of the connections that demonstrated a difference between HCs and PD patients emerged from the basal ganglia (Fig. 2). The highest number of affected connections (80%) emerged from the SN. The identified multivariate connectivity pattern included connections between the SN and the bilateral precentral gyrus, postcentral gyrus, precuneus, superior frontal gyrus, caudate, midbrain, pons, and medulla; between the LC and thalamus and the midbrain; and between the hippocampus and amygdala. The patterns obtained using diffusion‐based measures did not show the same pattern (Fig. S2‐S3). The correlation values of the LVs with age and gender (age: *r* = −0.133, *P* = 0.1547; gender: *r* = 0.088, *P* = 0.3473) do not suggest that they influenced the model. After subdividing the PD data into PDnonRBD and PDRBD, comparisons with the control group showed that the PDRBD group exhibited more affected connections with a decreased R1 between the basal ganglia and cortex, as well as more corticocortical connections. In contrast, when we directly compared the two PD groups, the PLS analysis did not identify any significantly different connection. This could be because of the small sample size of the PDRBD group.

**FIG 3:**
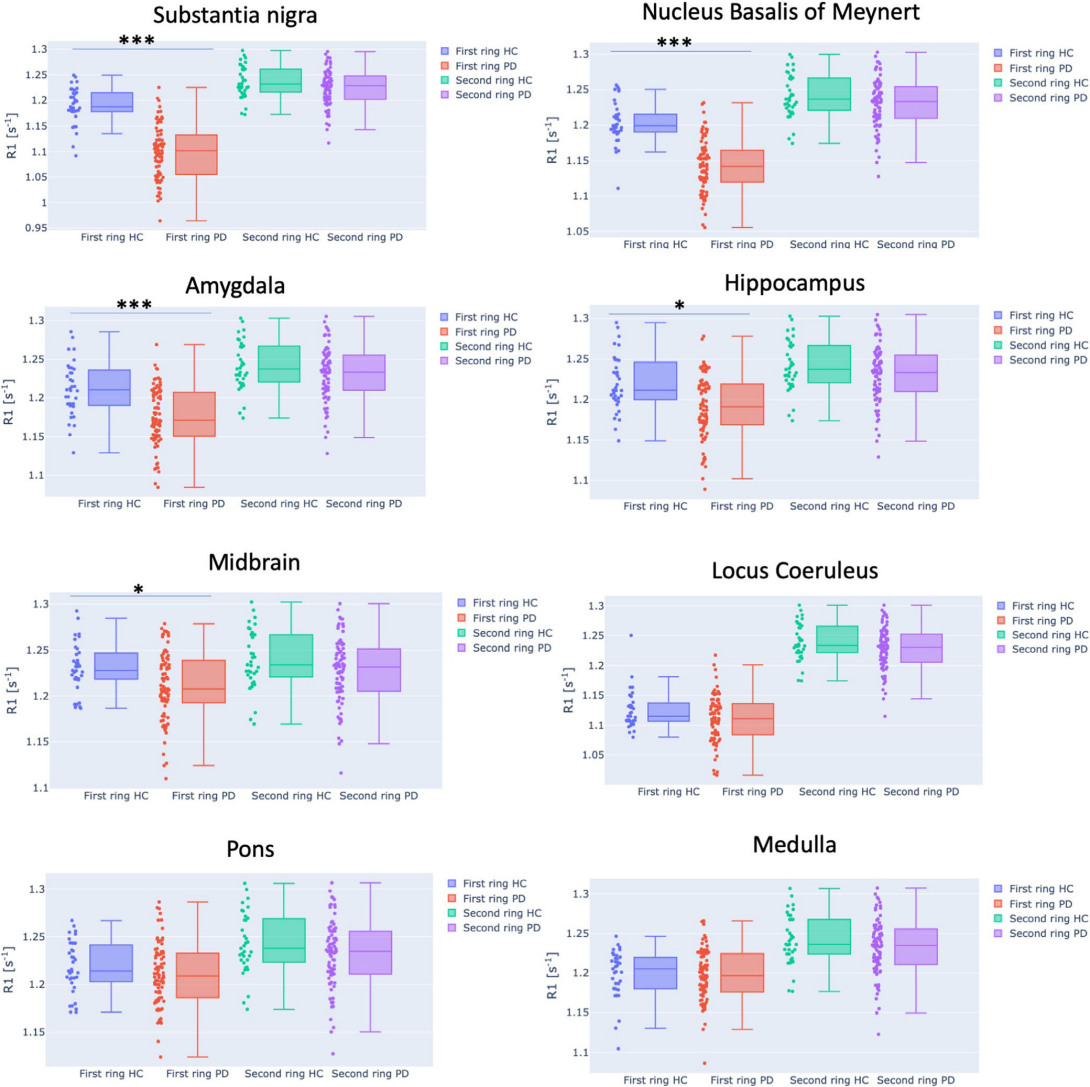
Ring analysis of the eight potential bilateral epicenter regions. A significant difference of the median longitudinal relaxation rate (R1) was found for the first rings of substantia nigra, hippocampus and amygdala, nucleus basalis of Meynert, and midbrain. No significant difference was observed for the second rings for all seven potential epicenters. Dots in the bar plots represent the subject's median R1 of the first and second rings, respectively. *** indicates a significant difference of the median R1 of the ring with P < 0.001, while * indicates a significant difference with a P < 0.05. [Color figure can be viewed at wileyonlinelibrary.com]

To further examine how the disease progression affected the myelin content, we performed a connectivity ring analysis in which the connections were subdivided into two sets (Fig. 3). Among the subcortical regions, all four regions emerged as potential epicenters: the SN, amygdala, hippocampus, and NBM. The median R1 of the SN first ring was significantly decreased in the PD groups compared with controls (*P* = 1.02 × 10^−11^, analysis of covariance corrected for multiple comparisons). Significant decreases of the first ring median R1 value were also obtained for the NBM (*P* = 6.98 × 10^−11^), amygdala (*P* = 1.22 × 10^−12^), and hippocampus (*P* = 0.0017). There was no significant difference between the HC and PD groups in the second ring's median R1.

Among the brainstem regions (midbrain, pons, medulla, and LC), we found a significant difference only for the midbrain's first ring median R1 between the HC and PD groups (*P* = 0.046), and that relationship was only marginally significant. We did not find any significant differences in the median R1 distributions within the epicenters.

**FIG 4:**
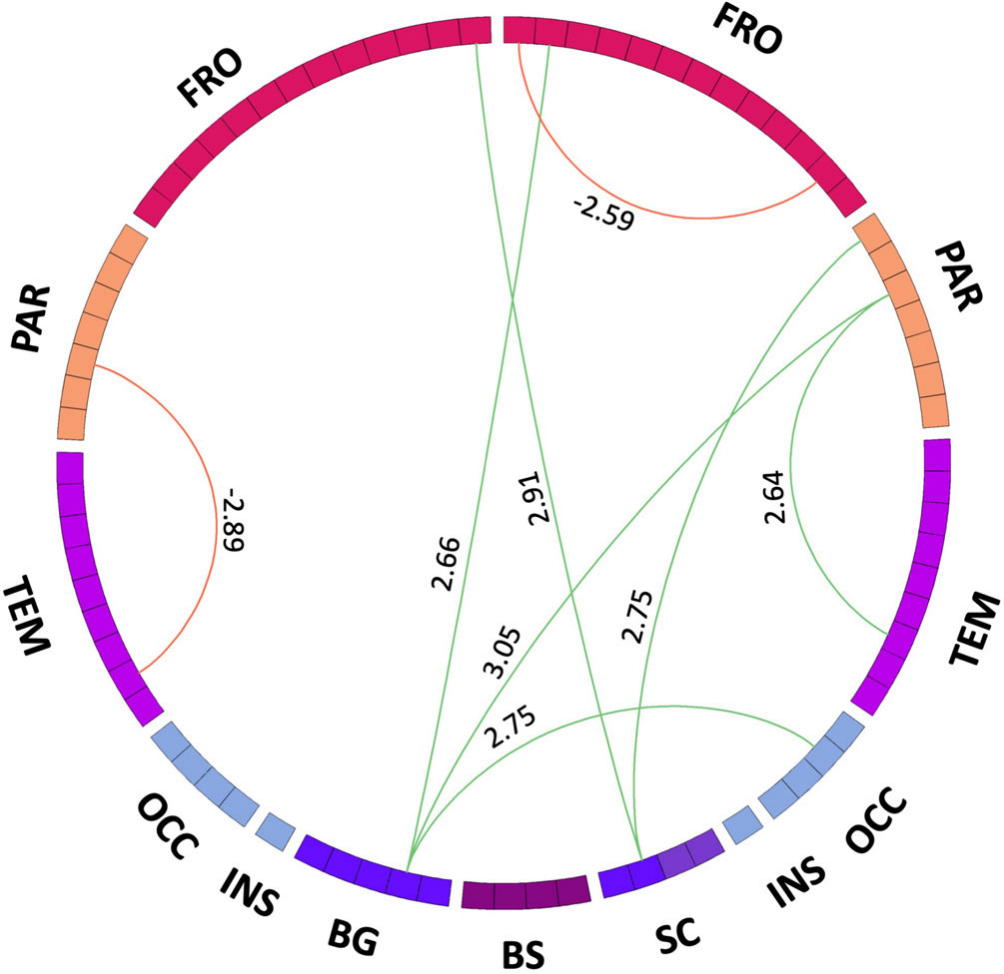
Multivariate connectivity pattern from the behavioral partial least squares that identifies the connections that maximally covary with the Movement Disorder Society Unified Parkinson's Disease Rating Scale (MDS‐UPDRS) Part III. The red links represent positive covariance with the clinical score, while the green ones represent negative covariance. The number associated with each connection represents the BSR, which shows how reliably the connections contribute to the multivariate connectivity pattern. BG, basal ganglia; BS, brainstem; FRO, frontal lobe; OCC, occipital lobe; PAR, parietal lobe; SC, subcortical regions, including amygdala, hippocampus, thalamus, and nucleus basalis of Meynert; TEM, temporal lobe. [Color figure can be viewed at wileyonlinelibrary.com]

To correlate these findings with clinical symptoms, we then examined the relationship of the myelin content with the clinical scores using behavioral PLS. We found that the MDS‐UPDRS Part III correlates positively with the identified multivariate connectivity pattern (*r* = −0.85, *P* < 0.01) (Fig. 4). For six of the eight connections identified, the LVs estimated with the behavioral PLS analysis negatively covaried with MDS‐UPDRS Part III clinical score. The connections that negatively covaried with the MDS‐UPDRS Part III score were between the putamen and precentral gyrus (BSR = 2.66), superior parietal gyrus (BSR = 3.05), and lateral occipital gyrus (BSR = 2.75), as well as between the thalamus and paracentral lobule (BSR = 2.9) and the postcentral gyrus (BSR = 2.75), and between the inferior temporal gyrus and superior parietal gyrus (BSR = 2.64). Two connections positively covaried with the MDS‐UPDRS Part III score: between inferior parietal gyrus and temporal pole (BSR = −2.89) and between paracentral lobule and rostral anterior cingulate gyrus (BSR = −2.59). We did not find any significant connectivity pattern for any of the other clinical scores.

## Discussion

In this study, we have shown that the myelin‐weighted connectome was able to identify alterations of the myelin content along connections that were mostly emerging from the basal ganglia. Using a ring analysis, we have shown that the SN, NBM, amygdala, hippocampus, and midbrain present significantly decreased R1 in the connections directly emerging from them and could be confirmed as potential epicenters. In addition, using behavioral PLS, we identified a subnetwork that maximally covaried with the MDS‐UPDRS Part III motor score. When performing behavioral PLS on the clinical scores associated with cognitive impairment, we could not identify any subnetwork significantly associated with those scores. This appears to suggest that cognitive deficits associated with demyelination are more subtle than the motor ones.

Most of the connections showing alterations in the myelin content between the PD and HC groups were emerging from the SN, connecting it with the caudate nucleus, frontal and parietal cortical areas, and the brainstem regions. This is in line with previous studies that have reported altered microstructural integrity along the nigrostriatal connections,^19^, ^71^, ^72^ as well as altered functional connectivity of the cortical regions connected to SN.^73^ Moreover, according to the Braak and Del Tredici hypothesis, the regions with long projections that are unmyelinated or poorly myelinated are more susceptible to Lewy body pathology.^5^ Because the nigrostriatal connections are poorly myelinated, it is expected that the SN is more susceptible to this pathology. Using R1 to probe a poorly myelinated region would result in a reduced dynamic range for the myelin measurement, but the robustness and high resolution of the MP2RAGE sequence produced reliable statistics.^74^, ^75^ Other affected connections were identified between the LC and thalamus and the LC and midbrain, as well as between the hippocampus and amygdala, which is also in line with the pathological lesions in the LC, as reported from a previous study that used neuromelanin‐sensitive MRI.^10^

Previous studies have reported that the PDRBD group has a more extensive form of PD compared with the PDnonRBD group.^76^ They found increased nodal measures (local efficiency, clustering coefficient, nodal betweenness, and nodal degree) in the limbic system and the neocortex in the PDRBD group compared with the PDnonRBD group. In line with those results, in our PLS analysis the multivariate connectivity pattern did not vary much when we analyzed the whole PD group compared with when we divided the patients into PDnonRBD and PDRBD groups. Nevertheless, the direct comparison between PDnonRBD and PDRBD did not lead to any significant results, so future work is needed to make further considerations on the differences between these subgroups.

We then tested the hypothesis that most of the connections emerging from the potential disease epicenters would have decreased myelin content compared with the HCs. Our results showed a significantly decreased median R1 along the connections emerging from the SN in the PD population compared with the control population. Previous imaging studies have already shown that the SN is an epicenter from where the disease spreads.^10^, ^12^, ^77^ Besides the SN, the amygdala, hippocampus, NBM, and midbrain have also shown significantly reduced R1 in their first ring. Because the first ring connections are directly connected with the related epicenters, this result suggests lower myelination in such connections, whereas the second ring, and therefore indirect, connections showed myelination comparable with HCs. This is consistent with the alternative “top‐down” model of PD progression, as reported in previous studies.^10^, ^11^ In contrast, previous studies have reported that the PDRBD follows the “bottom‐up” model of disease progression. However, we did not observe significant differences of the first ring's median R1 for the brainstem regions. This could be due to the fact that the whole‐brain tractography could not always reconstruct the fibers emerging from small structures, such as the LC, without anatomical priors on the fiber structure itself.^78^, ^79^ Another possibility is that the brainstem's larger structures, such as the pons and medulla, could be overrepresented with connections that are not affected, which may give misleading results.

Finally, using behavioral PLS, we identified a subnetwork of connections that maximally covaried with the MDS‐UPDRS Part III motor clinical score. Most of the identified connections negatively covaried with the motor clinical score and emerge from the putamen connecting it with the precentral and superior parietal gyrus. This is in line with the motor symptoms caused by the disease, because the putamen is linked to motor performance, as it has been shown previously.^80^ Other connections that also negatively covaried with the motor score were identified between the thalamus and the paracentral lobule and postcentral gyrus, which is also in agreement with previous diffusion and functional MRI studies.^72^, ^81^

There are several limitations to this study. First, R1 can be influenced by factors other than myelin, in particular the presence of edema or inflammation processes. Unfortunately, any MRI measure of myelin can be influenced by other phenomena, but a recent meta‐analysis showed that most MRI techniques are comparable when quantifying myelin content.^31^ Another factor is iron accumulation; because iron accumulation has been shown to take place in PD,^82^ we cannot exclude that it may have influenced these results, and further work is needed to explore this perspective. Second, our sample included an unbalanced number of PDRBD and PDnonRBD patients: we had a greater population of PDnonRBD compared with PDRBD, which could have driven the results when analyzing the whole PD population.

In conclusion, the myelin‐weighted connectome determined alterations in myelin content in PD patients, especially in connections emerging from the basal ganglia. Furthermore, the alterations of myelin content along the connections emerging from the potential epicenters are in line with the “top‐down” model of disease progression for PDnonRBD. Further investigation is needed to validate that the alterations of myelin content along the connections emerging from the small brainstem nuclei in PDRBD follow the “bottom‐up” model of disease progression.

## Author Roles

1. Research Project: (A) Conception, (B) Organization, (C) Execution;

2. Statistical Analysis: (A) Design, (B) Execution, (C) Review and Critique;

3. Manuscript: (A) Writing of the first draft, (B) Review and Critique.

T.B.: 1A, 1B, 1C, 2A, 2B, 2C, 3A, 3B

J.C.‐D.: 2C, 3B

B.M.: 1A, 1B, 2A, 2C, 3B

I.A.: 1A, 3B

J.‐C.C.: 1A, 2C, 3B

M.V.: 1A, 2C, 3B

S.L.: 1A, 1B, 1C, 2A, 2C, 3B

N.S.: 1A, 1B, 1C, 2A, 2B, 2C, 3A, 3B

M.M.: 1A, 1B, 1C, 2A, 2B, 2C, 3A, 3B

## Financial Disclosures

Tommy Boshkovski reports no disclosures. Julien Cohen‐Adad reports no disclosures. Bratislav Misic reports no disclosures. Isabelle Arnulf received consultancies from IDORSIA Pharma. Jean‐Christophe Corvol served on advisory boards for Air Liquide, Biogen, Biophytis, Denali, Ever Pharma, Idorsia, Prevail Therapeutic, Theranexus, and UCB and received grants from Sanofi and The Michael J. Fox Foundation. Marie Vidailhet received grants from Fondation d'Entreprise EDF and the Fondation Thérèse and René Planiol pour l'étude du Cerveau and unrestricted support for research on Parkinson's disease from Energipole (M. Mallart) and Société Française de Médecine Esthétique (M. Legrand). Stéphane Lehéricy received grants from Agence Nationale de la Recherche (ANRMNP 2009, Nucleipark), DHOS‐Inserm (2010, Nucleipark), France Parkinson (2008), Ecole Neuroscience de Paris, “Investissements d'avenir” (grants ANR‐10‐IAIHU‐06 and ANR‐11‐INBS‐0006) during the conduct of the study and a research grant from Biogen Inc. Nikola Stikov reports no disclosures. Matteo Mancini reports no disclosures.

## Supporting information


**Appendix S1:** Supporting Information.

## Data Availability

Data available on request from the authors
