# Supplementary material for: The Myelin‐Weighted Connectome in Parkinson's Disease
Source: Mov Disord. 2021 Dec 22;37(4):724–33. doi: 10.1002/mds.28891 (PMC9303520; doi:10.1002/mds.28891)
Supplement: Supplementary file 1 — Appendix S1: Supporting Information. [file MDS-37-724-s001.pdf]

Supplementary Materials

Brain atlas

Table S1. Brain ROIs

Location	ROI
Frontal	paracentral
Frontal	precentral
Frontal	caudalanteriorcingulate
Frontal	parsopercularis
Frontal	parstriangularis
Frontal	lateralorbitofrontal
Frontal	medialorbitofrontal
Frontal	frontalpole
Frontal	rostralmiddlefrontal
Frontal	caudalmiddlefrontal
Frontal	parsorbitalis
Frontal	rostralanteriorcingulate
Frontal	superiorfrontal
Parietal	postcentral

Parietal	posteriorcingulate
Parietal	superiorparietal
Parietal	supramarginal
Parietal	inferiorparietal
Parietal	isthmuscingulate
Parietal	precuneus
Temporal	fusiform
Temporal	parahippocampal
Temporal	superiortemporal
Temporal	transversetemporal
Temporal	bankssts
Temporal	entorhinal
Temporal	inferiortemporal
Temporal	temporalpole
Temporal	middletemporal
Occipital	cuneus
Occipital	lateraloccipital
Occipital	lingual

Occipital	pericalcarine
Insular	insula
Subcortical	Hippocampus
Subcortical	Amygdala
Subcortical	ThalamusProper
Subcortical	NucleusBasalis
Basal ganglia	Caudate
Basal ganglia	Putamen
Basal ganglia	Pallidum
Basal ganglia	AccumbensArea
Basal ganglia	SubstantiaNigra
Brainstem	LocusCoeruleus
Brainstem	Midbrain
Brainstem	Pons
Brainstem	Medulla

PLS Analysis

The partial least squares (PLS) analysis[1,2] is a multivariate statistical technique that tries to identify a linear combination of connections that maximally covary with an experimental desing or behavioral/clinical scores.

For each pair (HC vs. PD ; HC vs. PDnonRBD; HC vs. PDRBD), the two sets of variables were defined as $\mathbf{X}_{\mathbf{nxp}}$ and $\mathbf{Y}_{\mathbf{nx1}}$. $\mathbf{X}_{\mathbf{nxp}}$ corresponds to the non-zero connections shared between the groups, while $\mathbf{Y}_{\mathbf{nx1}}$ is a matrix that corresponds to the experiment design, where *n* corresponds to number of subjects, *p* corresponds to number of unique connections. The resulting sizes for the two sets of variables for each pair of groups was HC vs. PD: $X_{116x1360}$ and Y_{116x1}; HC vs PDnonRBD: $X_{81x1360}$ and Y_{81x1}; HC vs PDRBD: $X_{57x1349}$ and Y_{57x1}. Because the connectivity matrices are symmetric, we extracted the elements of the upper triangle that correspond to unique non-zero connections that are shared between the groups. We then stacked the unique connections on top of each other, i.e., each row of **X** corresponds to the unique connections of the subjects (both HC and PD group, HC and PDnonRBD, and HC and PDRBD), while **Y** contains the experimental design. For each pair of groups, a mean-centered matrix $\mathbf{M}^{\mathbf{dev}}_{\mathbf{2xp}}$, was then computed from **X** and **Y,** by removing the grand mean from **X** for each group as defined in **Y**, reflecting the covariation of each connection with the experimental design.

For the behavioural PLS, we aimed to identify the connections that maximally covaried with the MDS-UPDRS III score. The only difference from the mean-centing PLS is that the $\mathbf{Y}_{\mathbf{nx1}}$ contains the MDS-UPDRS III scores for each subject and the $\mathbf{M}^{\mathbf{dev}}_{\mathbf{2xp}} = \mathbf{Y'X}$.

Afterwards, a singular value decomposition (SVD) was applied to the $\mathbf{M}^{\mathbf{dev}}_{\mathbf{2xp}}$ matrix which resulted in a set of 2 mutually orthogonal latent variables (LVs):

$$\mathrm{SVD}(\mathbf{M}^{\mathbf{dev}}_{\mathbf{2xp}}) = \mathbf{U}_{px2}\, \mathbf{\Delta}_{2x2}\, \mathbf{V}_{2x2}'$$

where U and V are the left and right singular vectors, and Δ is a diagonal matrix with singular values along the diagonal. Each latent variable is composed of left and right singular vectors and a singular value. For example the first latent variable is composed of the left and right singular vectors (first column of U and V) reflecting the contribution of the and the singular value (first element of the diagonal matrix Δ). In our case the left singular vector provides the contribution of each connection to its respective LV, while the right singular vector reflects the contribution of the design variable to its respective LV and can be seen as a contrast. Furthermore, the singular value reflects the covariance between the two sets.

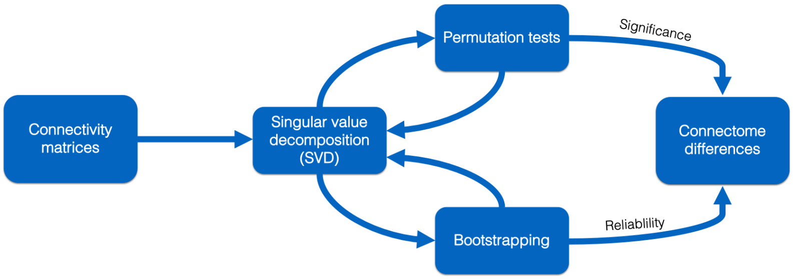

Figure S1. PLS analysis procedure

Ring-based analysis

Table S2. List of the regions belonging to the first ring for each epicenter and for at least 60% of the subjects in each group.

First Ring							
SN	NBM	Amygdala	Hippocampus	LC	Midbrain	Pons	Medulla
lateraloccipital	AccumbensArea	Hippocampus	AccumbensArea	Thalamus	Amygdala	AccumbensArea	Caudate
postcentral	Amygdala	Midbrain	Amygdala	Midbrain	Caudate	Amygdala	Nucleus Basalis
precentral	Caudate	Nucleus Basalis	Caudate	Pons	Hippocampus	Caudate	superiorfrontal
superiorfrontal	Hippocampus	Pons	Midbrain	Medulla	Nucleus Basalis	Hippocampus	
NucleusBasalis	Medulla	Putamen	Nucleus Basalis		lingual	NucleusBasalis	
Thalamus	Midbrain	Thalamus	Pallidum		middletemporal	Pallidum	
Caudate	Pallidum	fusiform	Pons		precentral	Putamen	
Amygdala	Pons	inferiortemporal	Putamen		superiorfrontal	Thalamus	
postcentral	Putamen	lateraloccipital	Thalamus		superiortemporal	isthmuscingulate	
precentral	SubstantiaNigra	lingual	cuneus			lateraloccipital	
precuneus	Thalamus	superiorparietal	entorhinal			postcentral	
superiorfrontal	fusiform	superiortemporal	fusiform			precuneus	
Pons	inferiortemporal	temporalpole	inferiorparietal			superiorfrontal	

	lateraloccipital<br>lingual<br>medialorbitofrontal<br>middletemporal<br>pericalcarine<br>precuneus<br>superiorfrontal<br>superiorparietal<br>superiortemporal<br>temporalpole		inferiortemporal<br>insula<br>isthmuscingulate<br>lateraloccipital<br>lingual<br>medialorbitofrontal<br>middletemporal<br>parahippocampal<br>pericalcarine<br>precuneus<br>superiorfrontal<br>superiorparietal<br>superiortemporal<br>temporalpole			superiorparietal	

Mean-centered PLS

Table S3. Multivariate connectivity pattern from the mean-centered PLS analysis between HC and PD Group. The reported labels correspond to the labels as defined in the Desikan-Killiany atlas.

SubstantiaNigra	postcentral_l
SubstantiaNigra	precentral_l
SubstantiaNigra	precuneus_l
SubstantiaNigra	superiorfrontal_l
SubstantiaNigra	superiorparietal_l
SubstantiaNigra	Caudate
SubstantiaNigra	precentral_r
SubstantiaNigra	precuneus_r
SubstantiaNigra	superiorfrontal_r
SubstantiaNigra	Midbrain
SubstantiaNigra	Pons
SubstantiaNigra	Medulla
ThalamusProper	LocusCoeruleus
Hippocampus	Amygdala
LocusCoeruleus	Midbrain

Table S4. Multivariate connectivity pattern from the mean-centered PLS analysis between HC and PDnonRBD group. The reported labels correspond to the labels as defined in the Desikan-Killiany atlas.

SubstantiaNigra	postcentral_l
SubstantiaNigra	precentral_l
SubstantiaNigra	precuneus_l
SubstantiaNigra	superiorfrontal_
SubstantiaNigra	superiorparietal_l
SubstantiaNigra	Caudate
SubstantiaNigra	postcentral_r
SubstantiaNigra	precentral_r
SubstantiaNigra	precuneus_r
SubstantiaNigra	superiorfrontal_r
SubstantiaNigra	Midbrain
SubstantiaNigra	Pons
SubstantiaNigra	Medulla
ThalamusProper	Amygdala
ThalamusProper	LocusCoeruleus
ThalamusProper	NucleusBasalis
Hippocampus	Amygdala
LocusCoeruleus	Midbrain
LocusCoeruleus	Pallidum

Table S5. Multivariate connectivity pattern from the mean-centered PLS analysis between HC and PDRBD group. The reported labels correspond to the labels as defined in the Desikan-Killiany atlas.

SubstantiaNigra	precuneus_l
SubstantiaNigra	isthmuscingulate_l
SubstantiaNigra	lateraloccipital_l
SubstantiaNigra	superiorfrontal_l
SubstantiaNigra	superiorparietal_l
SubstantiaNigra	Caudate
SubstantiaNigra	postcentral_r
SubstantiaNigra	precentral_r
SubstantiaNigra	precuneus_r
SubstantiaNigra	superiorfrontal_r
SubstantiaNigra	Midbrain
SubstantiaNigra	Pons
SubstantiaNigra	Medulla
ThalamusProper	NucleusBasalis
ThalamusProper	Amygdala
Hippocampus	postcentral_l
Hippocampus	Amygdala
Putamen	AccumbensArea
Midbrain	parsopercularis_r

Table S6. Mean and standard deviation of the median R1 distribution for each epicenter in the two groups. No significant difference was observed for any epicenter (i.e. all the p-values were equal to 1).

	HC	PD
SN	1.08 ± 0.027	1.15 ± 0.03
NBM	0.78 ± 0.02	0.82 ± 0.023
Hippocampus	0.7527 ± 0.016	0.79 ± 0.014
Amygdala	0.7596 ± 0.018	0.8 ± 0.017
LC	0.9917 ± 0.028	1.05 ± 0.027
Midbrain	1.0544 ± 0.025	1.12 ± 0.026
Pons	1.0968 ± 0.028	1.15 ± 0.026
Medulla	1.0352 ± 0.023	1.09 ± 0.019

DWI and NODDI processing

To complement the myelin weighted network, we also weighted the structural brain network with measures computed from the DWI data, specifically fractional anisotropy (FA), mean diffusivity (MD), and the intracellular volume fraction (ICVF), the former ones calculated using a tensor representation, the latter obtained using the neurite orientation dispersion and density imaging (NODDI) model[3]. The diffusion images were first preprocessed, with the same preprocessing pipeline as explained in the manuscript, to remove the known artifacts. Tensor-based measures were computed using MRtrix 3.0. Then, the preprocessed images were used to fit the NODDI model[3] using the open-source tool AMICO[4] (https://github.com/daducci/AMICO commit hash: 9fcb61b). The procedure to map ICVF to the tractogram is the same as for the R1. Afterwards, we performed the PLS analysis between the HC and the PD groups. The results are shown in Figures S2-S3.

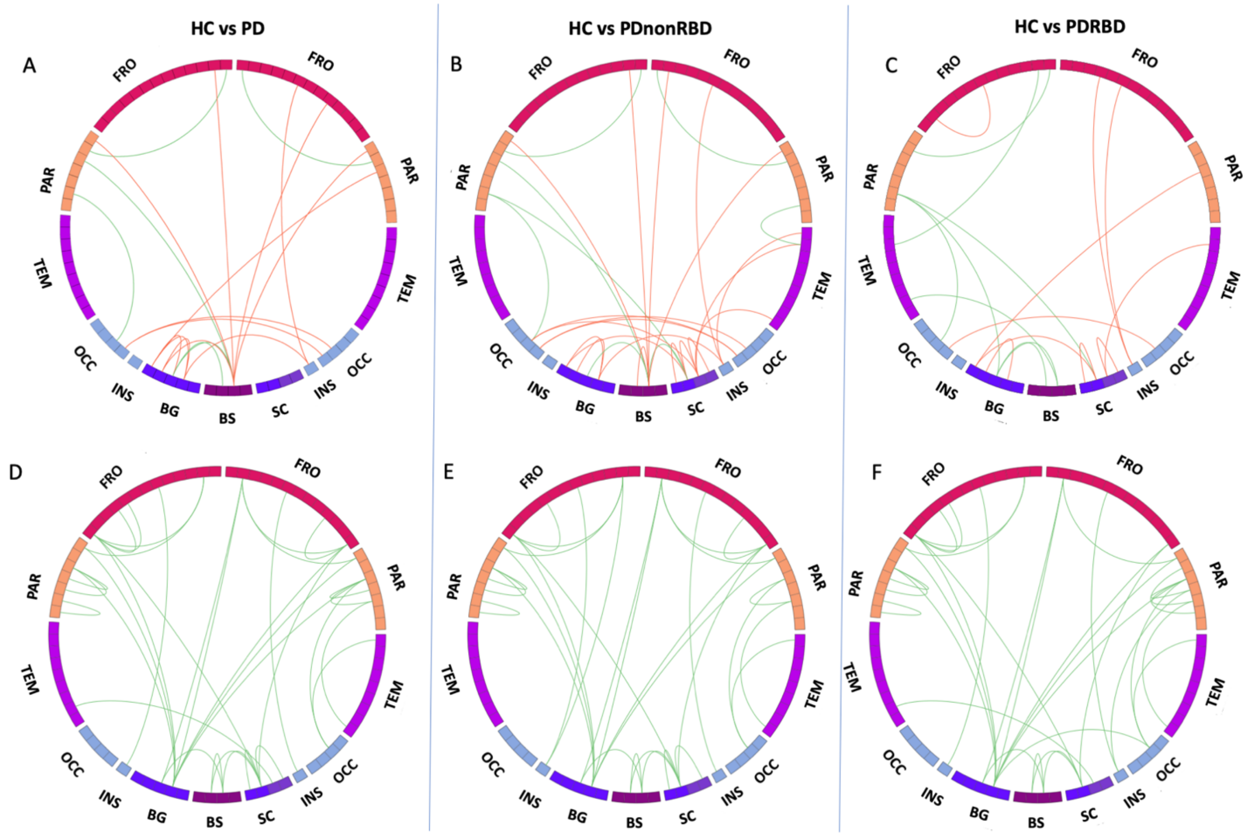

Figure S2. Mean-centering PLS of the FA (top row: A, B, C) and MD (bottom row: D, E, F). The red links correspond to higher metric (FA or MD) in HC compared to the patients, while the green links correspond to lower metric (FA or MD) in the HC compared to the patients. FRO (Frontal lobe), PAR (Parietal lobe), TEM (Temporal lobe), OCC (Occipital lobe), SC (Subcortical regions including: amygdala, hippocampus, thalamus, and nucleus basalis of Meynert), BG (Basal ganglia), BS (Brain stem).

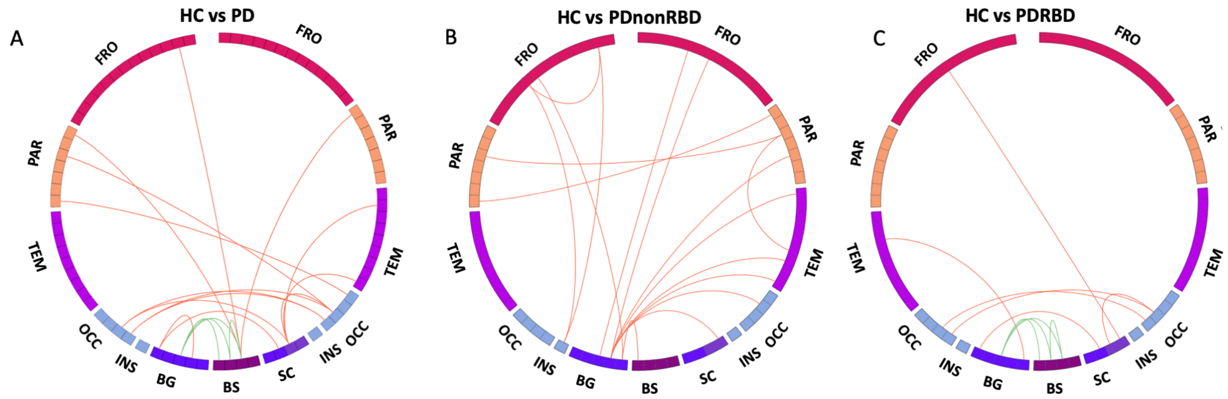

Figure S3. Connectogram of the multivariate connectivity pattern obtained with mean-centering PLS, composed of the connections that maximally covary between the groups. The connections in red showed decreased ICVF in the PD groups compared to HC, while the connections in green showed increased ICVF in the PD group compared to HC. FRO (Frontal lobe), PAR (Parietal lobe), TEM (Temporal lobe), OCC (Occipital lobe), SC (Subcortical regions including: amygdala, hippocampus, thalamus, and nucleus basalis of Meynert), BG (Basal ganglia), BS (Brain stem).

References

1. McIntosh AR, Lobaugh NJ. Partial least squares analysis of neuroimaging data: Applications and advances. In: *NeuroImage*. Vol 23. Academic Press; 2004:S250-S263. doi:10.1016/j.neuroimage.2004.07.020
2. Krishnan A, Williams LJ, McIntosh AR, Abdi H. Partial Least Squares (PLS) methods for neuroimaging: A tutorial and review. *Neuroimage*. 2011;56(2):455-475. doi:10.1016/j.neuroimage.2010.07.034
3. Zhang H, Schneider T, Wheeler-Kingshott CA, Alexander DC. NODDI: Practical in vivo neurite orientation dispersion and density imaging of the human brain. *Neuroimage*. 2012;61(4):1000-1016. doi:10.1016/J.NEUROIMAGE.2012.03.072
4. Daducci A, Canales-Rodríguez EJ, Zhang H, Dyrby TB, Alexander DC, Thiran JP. Accelerated Microstructure Imaging via Convex Optimization (AMICO) from diffusion MRI data. *Neuroimage*. 2015;105:32-44. doi:10.1016/J.NEUROIMAGE.2014.10.026
